# Influences of two coexisting endosymbionts, CI‐inducing *Wolbachia* and male‐killing *Spiroplasma*, on the performance of their host *Laodelphax striatellus* (Hemiptera: Delphacidae)

**DOI:** 10.1002/ece3.5392

**Published:** 2019-06-24

**Authors:** Kazuki Yoshida, Sachiyo Sanada‐Morimura, Shou‐Horng Huang, Makoto Tokuda

**Affiliations:** ^1^ Faculty of Agriculture Saga University Saga Japan; ^2^ Kyushu Okinawa Agricultural Research Center NARO Kumamoto Japan; ^3^ Chiayi Agricultural Experiment Station, Taiwan Agricultural Research Institute Council of Agriculture Chiayi Taiwan

**Keywords:** cytoplasmic incompatibility, endosymbiont, male‐killing, small brown planthopper, *Spiroplasma*, symbiosis, *Wolbachia*

## Abstract

The small brown planthopper *Laodelphax striatellus* (Hemiptera: Delphacidae) is reported to have the endosymbiont *Wolbachia*, which shows a strong cytoplasmic incompatibility (CI) between infected males and uninfected females. In the 2000s, female‐biased *L. striatellus* populations were found in Taiwan, and this sex ratio distortion was the result of male‐killing induced by the infection of another endosymbiont, *Spiroplasma*. *Spiroplasma* infection is considered to negatively affect both *L. striatellus* and *Wolbachia* because the male‐killing halves the offspring of *L. striatellus* and hinders the spread of *Wolbachia* infection via CI. *Spiroplasma* could have traits that increase the fitness of infected *L. striatellus* and/or coexisting organisms because the coinfection rates of *Wolbachia* and *Spiroplasma* were rather high in some areas. In this study, we investigated the influences of the infection of these two endosymbionts on the development, reproduction, and insecticide resistance of *L. striatellus* in the laboratory. Our results show that the single‐infection state of *Spiroplasma* had a negative influence on the fertility of *L. striatellus*, while the double‐infection state had no significant influence. At late nymphal and adult stages, the abundance of *Spiroplasma* was lower in the double‐infection state than in the single‐infection state. In the double‐infection state, the reduction of *Spiroplasma* density may be caused by competition between the two endosymbionts, and the negative influence of *Spiroplasma* on the fertility of host may be relieved. The resistance of *L. striatellus* to four insecticides was compared among different infection states of endosymbionts, but *Spiroplasma* infection did not contribute to increase insecticide resistance. Because positive influences of *Spiroplasma* infection were not found in terms of the development, reproduction, and insecticide resistance of *L. striatellus*, other factors improving the fitness of *Spiroplasma*‐infected *L. striatellus* may be related to the high frequency of double infection in some *L. striatellus* populations.

## 1 INTRODUCTION

Heritable bacterial endosymbionts are commonly found in diverse arthropods including insects (Bourtzis & Miller, 2006; Riegler & O'Neill, 2007). Some of them manipulate their host reproductive systems to increase their own fitness. The manipulation strategies increase the number of infected females in the population because the bacteria are fundamentally maintained by vertical transmission through egg cytoplasm (Ferree et al., 2005; Moran & Wernegreen, 2000). Such reproductive manipulations have evolved in phylogenetically diverse groups of bacteria, including *Wolbachia*, *Rickettsia*, *Arsenophonus*, *Spiroplasma*, and *Cardinium* (Moran, McCutcheon, & Nakabachi, 2008). At present, the following four types of reproductive manipulations have been commonly observed: (a) cytoplasmic incompatibility (CI) in matings between infected males and uninfected females (Poinsot, Charlat, & Mercot, 2003; Werren, 1997), (b) male‐killing, (c) thelytokous parthenogenesis, and (d) feminization of immature males (Stouthamer, Breeuwer, & Hurst, 1999).

In insect species, male‐killings are caused by various bacterial symbionts (Hurst, Jiggins, & Majerus, 2003), for example, *Wolbachia* in butterflies (Dyson, Kamath, & Hurst, 2002; Hurst, Jiggins, et al., 1999; Jiggins, Hurst, Schulenburg, & Majerus, 2001; Mitsuhashi, Fukuda, Nicho, & Murakami, 2004) and coleopterans (Fialho & Stevens, 2000; Hurst, Jiggins, et al., 1999), *Rickettsia* in coleopterans (Hurst, Majerus, & Walker, 1993; Lawson, Mousseau, Klaper, Hunter, & Werren, 2001; Werren et al., 1994), and *Arsenophonus* in a parasitoid wasp (Gherna et al., 1991; Werren, Skinner, & Huger, 1986). *Spiroplasma* is a well‐known male‐killer of diverse insect species, such as fruit flies *Drosophila* spp. (Montenegro, Solferini, Klaczko, & Hurst, 2005; Poulson & Sakaguchi, 1961; Williamson et al., 1999), a butterfly (Jiggins, Hurst, Jiggins, Schulenburg, & Majerus, 2000), ladybird beetles (Hurst, Schulenburg, et al., 1999; Majerus, Schulenburg, Majerus, & Hurst, 1999; Tinsley & Majerus, 2006), and a green lacewing (Hayashi, Watanabe, Yukuhiro, Nomura, & Kageyama, 2016).

Male‐killing is roughly divided into two patterns, early and late. In the former, it occurs at embryonic or early larval periods and in the latter at the mature larval stage. Early male‐killing has been suggested to contribute to the adaptive benefit of maternally inherited endosymbionts by the following presumed mechanisms: reallocation of resources from males to females, prevention of inbreeding and reduction of cannibalism among females (Hurst & Majerus, 1993; Hurst, 1993). Recently, a protein inducing early male‐killing was identified in *Drosophila*‐associated *Spiroplasma* (*S. poulsonii*) by Harumoto and Lemaitre (2018). The other type, late male‐killing, is rather rare and is reported only in a few species including mosquitoes by microsporidian (Andreadis, 1985; Andreadis & Hall, 1979), oriental tea tortrix moth by an unknown agent (Morimoto, Nakai, Ono, & Kunimi, 2001; Nakanishi, Hoshino, Nakai, & Kunimi, 2008), and *Drosophila* by *Spiroplasma* (Kageyama, Anbutsu, Shimada, & Fukatsu, 2007). Late male‐killing may be better strategy than early male‐killing if horizontal transmissions occur as in the case of microsporidian associated with mosquitoes (Dunn & Smith, 2001). Although male‐killing phenotypes should not be classified by the timing of male‐killing alone, the classification is important to understand the evolution and mechanism of male‐killing by endosymbionts.

Such host–endosymbiont relationships are very important to study the coevolution and cospeciation of organisms (Herre, Knowlton, Mueller, & Rehner, 1999; Hosokawa, Kikuchi, Nikoh, Shimada, & Fukatsu, 2006; Moran et al., 2008). For instance, if an endosymbiont increases the fitness of its host, it is also beneficial for the endosymbiont because its population density will be expected to increase. In this case, their relationship is mutualistic and may evolve into a closer one. Conversely, if the endosymbiont has negative influences on its host's fitness, the relationship is antagonistic and may facilitate the evolution of host characteristics to resist the endosymbiont.

In addition, interactions between different endosymbionts sharing the host are interesting study subjects to consider the coevolution of the host and endosymbionts. The coexistence of multiple endosymbiotic strains or species in a host is well known in various insect species (Buchner, 1965; Gómez‐Valero et al., 2004; Goto, Anbutsu, & Fukatsu, 2006; Kikuchi & Fukatsu, 2003; Kondo, Shimada, & Fukatsu, 2005; Skaljac, Zanic, Ban, Kontsedalov, & Ghanim, 2010; Thao et al., 2000; Thao, Gullan, & Baumann, 2002). The coexisting symbionts may compete for available resources and spaces in the host body or may share them. Besides, if a symbiont has some strategy to manipulate the host's physiology and/or behavior, the manipulation may also influence the density of coexisting endosymbionts. Limited environments, such as host bodies, must facilitate various interactions among the coexisting symbionts.

**Figure 1:**
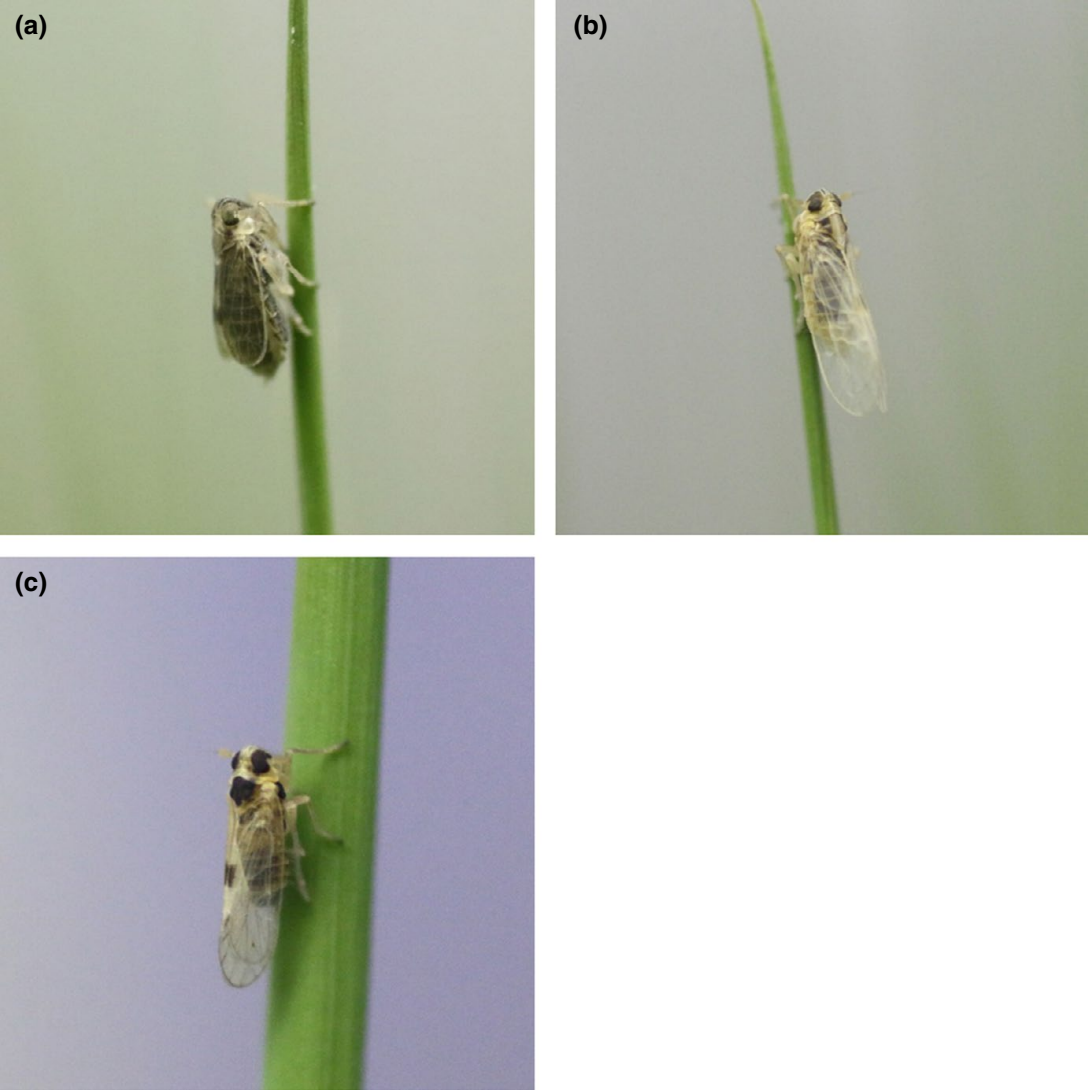
*Laodelphax striatellus*. Short‐winged female (a), long‐winged female (b), and long‐winged male (c)

The small brown planthopper *Laodelphax striatellus* (Fallen; Hemiptera: Delphacidae; Figure 1) is reported to have an endosymbiont *Wolbachia*, which exhibits strong CI between infected males and uninfected females (Noda, 1984a; Noda, Koizumi, Zhang, & Deng, 2001). In the 2000s, female‐biased *L. striatellus* populations were found in Taiwan and the sex ratio distortion was caused by male‐killing owing to the infection of another endosymbiont, *Spiroplasma* (Sanada‐Morimura, Matsumura, & Noda, 2013). Because the female‐biased populations in Taiwan were also infected with *Wolbachia*, this is a rare example of two endosymbionts possessing different manipulation strategies coexisting in the same insect population.

In this system, male‐killing caused by *Spiroplasma* may virtually invalidate the CI strategy of *Wolbachia*. Also, the offspring of *L. striatellus* are expected to reduce by half owing to male‐killing. Hence, the existence of *Spiroplasma* seems to be a disadvantage for both *L. striatellus* and *Wolbachia*. Even so, coinfection of *Wolbachia* and *Spiroplasma* was maintained at high frequencies in some areas of Taiwan (Sanada‐Morimura et al., 2013). This implies that *Spiroplasma* has some traits that increase the fitness of the host and/or coexisting organisms.

To reveal the interactions among *L. striatellus*, *Wolbachia*, and *Spiroplasma*, we investigated the influences of two symbionts on the developmental and reproductive performance of *L. striatellus*.

## 2 MATERIALS AND METHODS

### 2.1 Insects

**Figure 2:**
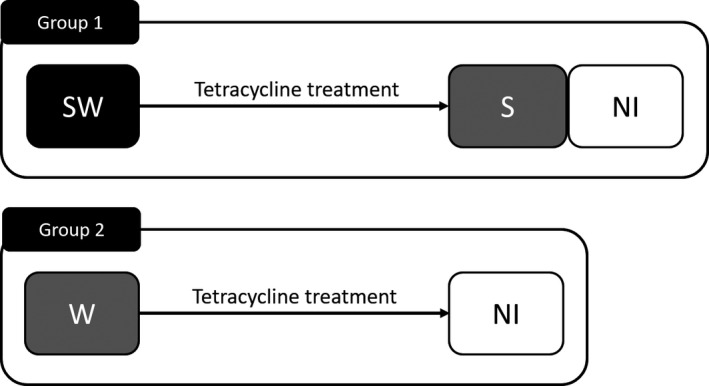
Schemes of antibiotic treatments to establish *Laodelphax striatellus* lines with different infection states: infected with both *Spiroplasma* and *Wolbachia* (SW), only with *Spiroplasma* (S) or *Wolbachia* (w) and noninfected (NI). The W line could not be created from the SW line by antibiotics (Sanada‐Morimura et al., 2013; authors' unpublished data). Group 1 consists of three lines with the same genetic background, the SW, S and NI lines. Group 2 includes the W line and NI_T‐7_ line originated from the W line

A strain of *L. striatellus* infected with both *Wolbachia* and *Spiroplasma* (SW line), which was established from a population of the eastern part of Taiwan in Sanada‐Morimura et al. (2013), was used in this study. From this strain, lines with different infection states, that is, the S line with single infection of *Spiroplasma* and the NI line with no infection of either endosymbiont, were established by tetracycline treatment. A detailed method is described in the next section (antibiotic treatments). Because *Wolbachia* single‐infected lines were not obtained from tetracycline (Sanada‐Morimura et al., 2013) and antibiotics (doxycycline, gentamicin, and chloramphenicol), a *Wolbachia* single‐infection strain (W line) originated from the eastern part of Taiwan (T‐7) was used. Then, another no infection line was established from the W line via tetracycline treatment (=NI_T‐7_ line; Figure 2). These strains were reared using rice seedlings (var. Reihou) at 25°C under 16L:8D photoperiodic conditions. In the SW and S lines, males are completely eliminated due to late male‐killing by *Spiroplasma* (Sanada‐Morimura et al., 2013) So, males must be supplied from other lines to continue the generations. For this purpose, males of NI_T‐7_ line were used to maintain these lines. Then, as mentioned earlier, strong CI occurs between *Wolbachia*‐infected males and *Wolbachia*‐uninfected females in our system (so the W line males cannot use for maintaining the S line.).

### 2.2 Antibiotic treatments

First‐instar nymphs of the SW line were fed rice seedlings treated with antibiotic solutions at different concentrations (0.005%, 0.01%, 0.05%, and 0.1%) in 15‐ml tubes. The antibiotic‐treated seedlings were changed every week until adult emergence. After that, females were individually transferred to glass tubes (2 cm diameter and 17 cm high) with rice seedlings, each paired with a male of the NI_T‐7_ line and allowed to lay eggs into the seedlings. Their progenies (F_1_) were reared on rice seedlings without tetracycline. DNA was extracted from F_1_ planthoppers, and bacterial infection was confirmed by PCR following the method described in Sanada‐Morimura et al. (2013). The W line was treated with only 0.05% tetracycline solution to establish the NI_T‐7_ line.

### 2.3 Comparisons of nymphal duration, adult longevity, and fertility among *Laodelphax striatellus* lines with different infection states

Nymphal duration, adult longevity, and fertility of *L. striatellus* were compared among lines with different infection states. In the analyses, the performance of SW, S, and NI lines (Group 1) was first compared to reveal the influence of *Spiroplasma* under the presence or absence of *Wolbachia*. Next, the performance of the W and NI_T‐7_ lines (Group 2) was compared to confirm the influence of *Wolbachia* infection (Figure 2).

First‐instar nymphs were individually introduced into 15‐ml tubes with three rice seedlings on hatching day and reared until adult emergence. Nymphal duration was recorded for each individual.

Each emerged female was transferred to a glass tube (2 cm diameter and 17 cm high) with an NI_T‐7_ male and five rice seedlings. The seedlings were exchanged twice a week, and old ones were stored in 70% ethanol. To evaluate female adult longevity, this procedure was continued until the death of each female. Eggs deposited into seedlings were counted under a stereoscopic microscope.

### 2.4 Quantifications of *Spiroplasma* densities in different ages of nymphs and adults of *Laodelphax striatellus*

Female adults of SW and S females were individually reared and paired with a male adult of NI_T‐7_ in a glass tube with rice seedlings for a week. Then, the seedlings were washed with water after removing adults and were maintained until the eclosion of eggs. Three days after the first hatching of offspring, some nymphs were collected and stored in 99.5% ethanol. Other nymphs were continuously reared with new rice seedlings. Some nymphs (up to 36 individuals) were collected every 3 days until 12 days from hatching, that is, samples of 0–3‐day‐old, 3–6‐day‐old, 6–9‐day‐old and 9–12‐day‐old nymphs were prepared. Adult samples were collected 17 days after hatching (=14–17‐day‐old). Total DNA was extracted individually from whole bodies using a DNeasy Blood & Tissue Kit (Qiagen). DNA of 0–3 and 3–6‐day‐old nymphs was extracted with a half volume of elution buffer of standard because they were tiny, and we expected concentrations of DNA to be very little. Nevertheless, the DNA concentration of *Spiroplasma* was too low to calculate in the 0–3‐day‐old nymphs. Therefore, 3–6‐day‐old nymphs and later stages were used for the comparisons.

Infection densities of *Spiroplasma* were individually determined by real‐time PCR with an absolute quantification method and compared between the SW and S lines in day‐old samples. Real‐time PCR was conducted with a quantitative PCR analyzer (LightCycler 480; Roche Diagnostics) using the LightCycler 480 SYBR Green I Master. Thermal conditions in RT‐qPCR were the same as those described by Sanada‐Morimura et al. (2013). The primer set of Sanada‐Morimura et al. (2013) was used for amplification of the DNA fragment of the RNA polymerase beta subunit gene from *Spiroplasma* as the target gene (forward; SpRpo2F: 5′‐AATGGTGGTGCTGGAATTGTTC‐3′, reverse; SpRpo2R: 5′‐TCCGTGTCTTCCAGCCATTT‐3′) and used for the fragment of the actin gene of the host insect as the reference gene (forward; LSactin1F: 5′‐AAGGACCTGTACGCCAACAC‐3′, reverse; LSactin1R: 5′‐GATCCTCCGATCCAAACAGA‐3′). The standard curve of the DNA concentration of *Spiroplasma* correlated with cycle threshold (Ct) was drawn using real‐time PCR amplicon of the target region after purification with the Amicon Ultra Centrifugal Filter (30K; Merck Millipore Ltd.) at concentrations of 10^4^, 10^5^, 10^6^, 10^7^, 10^8^, 10^9^, and 10^10^‐fold dilutions. Concentrations of DNA in these dilutions were calculated by the concentration of a 10‐fold dilution determined with spectrophotometer (NanoDrop Lite; Thermo Scientific). Amplification efficiency was 91.2% in a range of Ct from approximately 9 to 31; therefore, all quantification analyses of *Spiroplasma* were conducted within this range.

### 2.5 Insecticide resistance of *Laodelphax striatellus* with different infection states

Insecticide resistance of *L. striatellus* with the different infection states SW, S, W, and NI_T‐7_ was determined using a standard topical application method (Fukuda & Nagata, 1969) with four insecticides, BPMC (Sumitomo Chemical Co. Ltd.), imidacloprid (Bayer Crop Science K. K.), and etofenprox and dinotefuran (Mitsui Chemicals, Inc.). The dinotefuran test was conducted three times using different generations to confirm reproductivity. Although the SW and S lines and W and NI_T‐7_ lines are all originated from eastern parts of Taiwan, they do not have identical genetic backgrounds. Therefore, to compare these four lines directly, we conducted the insecticide tests after we approximated the background; that is, as shown in the above section (Insects), the crossbreedings of NI_T‐7_ males and SW and S females were continued more than 12 generations.

*Laodelphax striatellus* has wing dimorphism (short‐winged or long‐winged; Figure 1), which is determined by environmental and genetic factors (Kishimoto, 1956; Mori & Nakasuji, 1990). High population density especially promotes the production of long‐winged morphs. Long‐winged females were used for the comparisons of insecticide resistance in most previous studies, because they are generally dominant under laboratory‐rearing condition due to high population density in rearing cages. However, in this study, short‐winged morph is major type in the SW and S lines, probably because of relatively low population densities owing to the male‐killing in these lines. So we used short‐winged females for the insecticide resistance test. Short‐winged adult females within 7 days of emergence were anesthetized with CO_2_ gas for at least 5 s prior to the insecticide treatment. A 0.08 µl acetone solution of an insecticide was applied topically to the surface of insect body, except the legs and wings, with a hand micro‐applicator (Burkard Manufacturing Company Ltd.). The treated insects were kept in a transparent plastic cup (5 cm diameter, 10 cm high) with rice seedlings. Mortality was determined 24 hr after treatment for all insecticides. More than 45 individuals were tested at each insecticide concentration, and 5–8 different concentrations were tested. Average body weight was calculated for approximately 15 individuals in each infection state population at each insecticide test to convert the unit of LD_50_ from µg/insect to µg/g. The LD_50_ values, 95% confidence intervals, and the slopes of the regression lines were calculated by Bliss's (1935) probit method with PoloPlus software (LeOra software, 2003).

### 2.6 Statistics

Nymphal duration was analyzed by a generalized linear model (GLM) with a Poisson distribution and a log link, and adult longevity and fertility were assessed by GLM with a negatively binomial distribution using the glm.nb function in the MASS package. Infection densities of *Spiroplasma* were compared by GLM with a gamma distribution and an inverse link. In the analyses of Group 1, treatment means were compared by Tukey's HSD test. Statistical analyses were performed by R ver. 3.5.2 (R Core Team, 2018).

Insecticide resistances between different infection states were compared by likelihood ratio (LR) tests of equality for slopes and intercepts of probit regression lines. LR tests of equality were only conducted on slopes of probit regression lines. These analyses were performed by PoloPlus software (Robertson, Jones, Olguin, & Alberts, 2017). The significance level of each test was adjusted using the sequential Bonferroni method to control type I error across pairwise comparisons (Rice, 1989).

## 3 RESULTS

### 3.1 Development and reproduction of *Laodelphax striatellus* with different infection states

**Figure 3:**
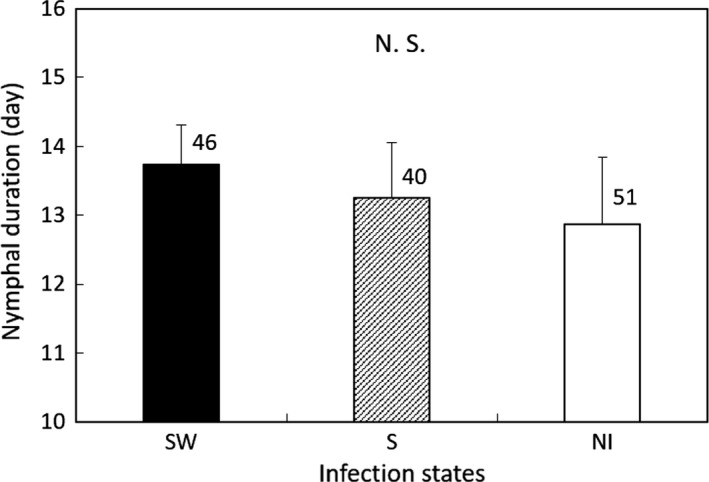
Female nymphal duration of the *Laodelphax striatellus* lines in Group 1 infected with both *Spiroplasma* and *Wolbachia* (SW), infected with only *Spiroplasma* (S) and noninfected (NI). Error bars indicate standard deviations. Numerals above columns are sample numbers. No significant differences were detected between infection states (*p* > 0.05; GLM followed by Tukey's HSD test)

**Figure 4:**
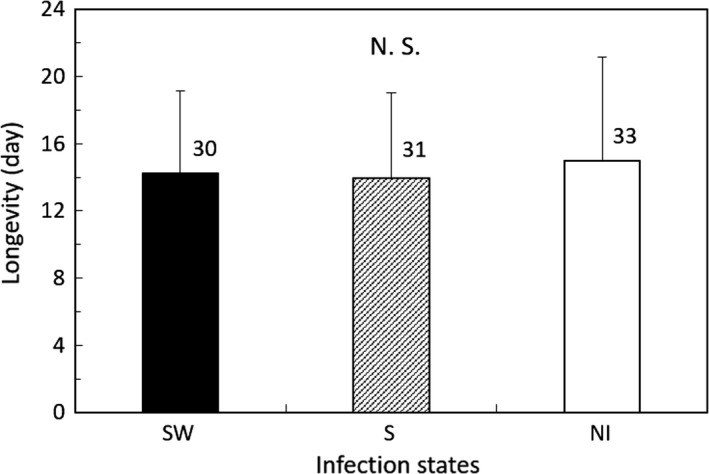
Female adult longevity of the *Laodelphax striatellus* lines in Group 1 infected with both *Spiroplasma* and *Wolbachia* (SW), infected with only *Spiroplasma* (S) and noninfected (NI). Error bars indicate standard deviations. Numerals above columns are sample numbers. No significant differences were detected between infection states (*p* > 0.05; GLM followed by Tukey's HSD test)

**Figure 5:**
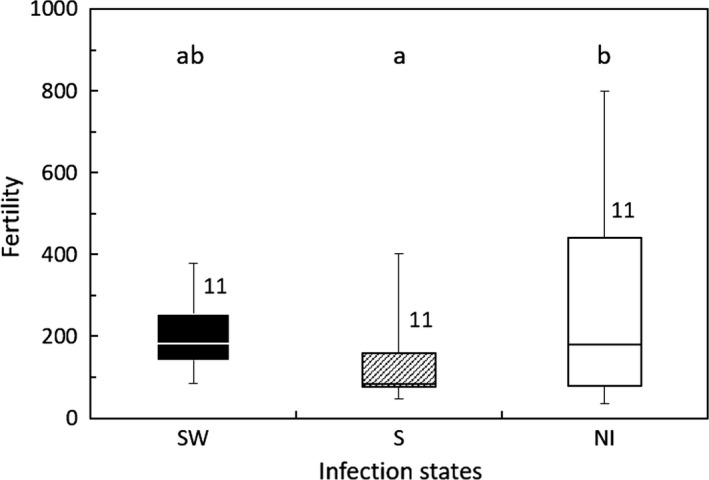
Fertility of the *Laodelphax striatellus* lines in Group 1 infected with both *Spiroplasma* and *Wolbachia* (SW), infected with only *Spiroplasma* (S) and noninfected (NI). Error bars indicate the range of data. Numerals above boxes are sample numbers. The same letters above boxes indicate no significant differences between infection states (*p* > 0.05; GLM followed by Tukey's HSD test)

In Group 1, nymphal duration and female adult longevity were not significantly different among the three lines (GLM; *df* = 2, *χ* ^2^ = 1.399, *p* = 0.497 for nymphal duration; *df* = 2, *χ* ^2^ = 0.620, *p* = 0.733 for female adult longevity; Figures 3 and 4). The fertility of the S line was lower than that of the NI line (GLM; *df* = 2, *χ* ^2^ = 6.707, *p* = 0.035; Tukey's HSD test, *z* = −2.60 and *p* = 0.389 between the NI and SW strain, and *z* = 1.291 and *p* = 0.400 between the S and SW strain; Figure 5).

**Figure 6:**
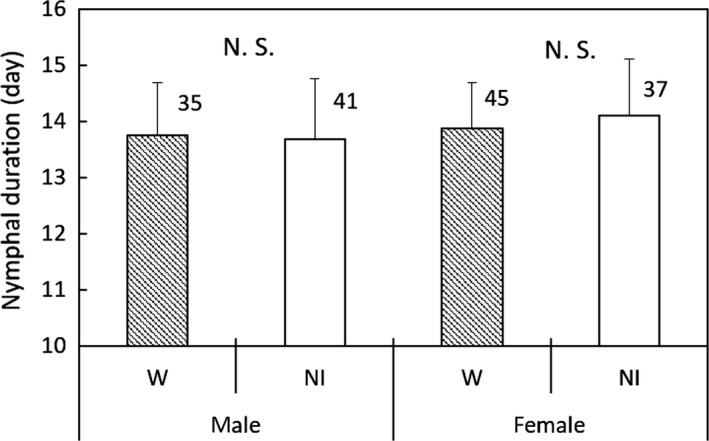
Nymphal duration of the *Laodelphax striatellus* lines in Group 2 infected with *Wolbachia* (W) and noninfected (NI). Error bars indicate standard deviations. Numerals above columns are sample numbers. No significant differences were detected between infection states in either sex (*p* > 0.05; GLM)

**Figure 7:**
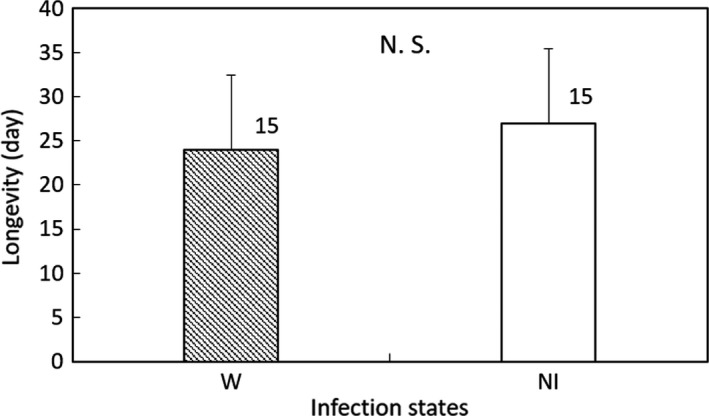
Female adult longevity of *Laodelphax striatellus* lines in Group 2 infected with *Wolbachia* (W) and noninfected (NI). Error bars indicate standard deviations. Numerals above columns are sample numbers. No significant differences were detected between infection states (*p* > 0.05; GLM)

**Figure 8:**
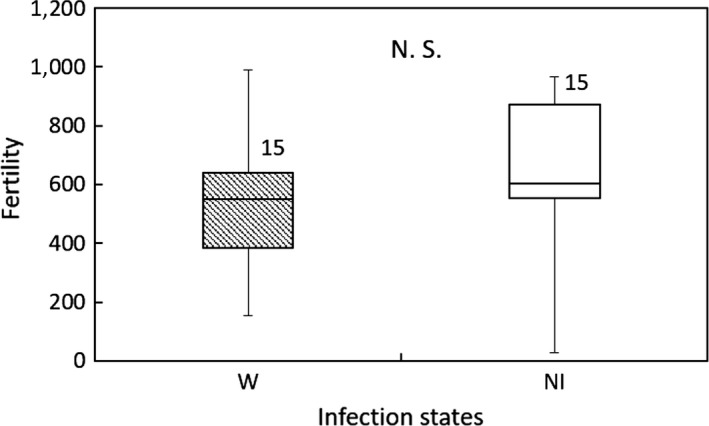
Fertility of the *Laodelphax striatellus* lines in Group 2 infected with *Wolbachia* (W) and noninfected (NI). Error bars indicate the range of data. Numerals above columns are sample numbers. No significant differences were detected between infection states (*p* > 0.05; GLM)

In Group 2, significant differences were not found between the W and NI_T‐7_ lines in nymphal duration (GLM; *df* = 1, *χ* ^2^ = 0.0846, *p* = 0.771 for female; *df* = 1, *χ* ^2^ = 0.0049, *p* = 0.944 for male; Figure 6), adult longevity (GLM; *df* = 1, *χ* ^2^ = 0. 858, *p* = 0.354; Figure 7), or fertility (GLM; *df* = 1, *χ* ^2^ = 0.620, *p* = 0.431; Figure 8).

### 3.2 *Spiroplasma* density between the single‐ and double‐infection lines of *Laodelphax striatellus*

**Figure 9:**
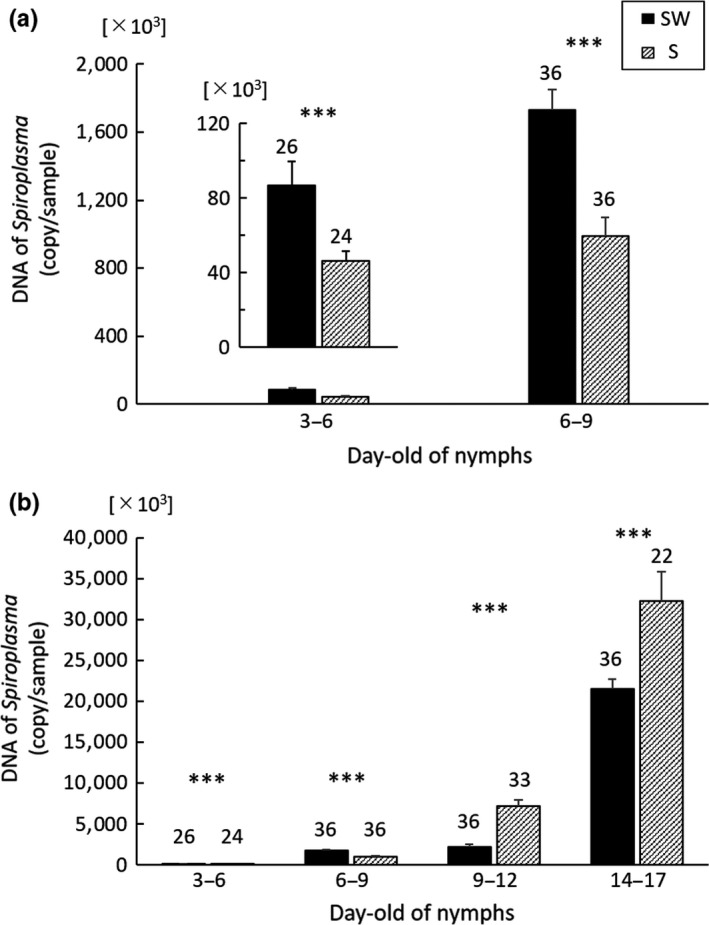
*Spiroplasma* infection densities of two *Laodelphax striatellus* lines (infected with both *Spiroplasma* and *Wolbachia* [SW] and with only *Spiroplasma* [S]) in 3–6 and 6–9‐day‐old nymphs (a), 3–6‐day‐old, 6–9‐day‐old and 9–12‐day‐old nymphs and adults (14–17 days from hatching) (b). The number of copies in a fragment of the internal region of the RNA polymerase gene from *Spiroplasma* in each sample was determined by real‐time quantitative PCR after calibration by the *actin* gene from *Laodelphax striatellus*. Error bars indicate standard errors. Numerals above columns are sample numbers. Asterisks indicate the significant differences of the DNA copies between the two lines at the same number of days old (***p < 0.001; GLM)

Although the *Spiroplasma* density of the SW line was higher than the S line in 3–6 and 6–9‐day‐old nymphs (GLM; *df* = 1, *χ* ^2^ = 11.06, *p* < 0.001 for 3–6‐day‐old nymphs; *df* = 1, *χ* ^2^ = 18.43, *p* < 0.001 for 6–9‐day‐old nymphs; Figure 9a), the density was lower in 9–12‐day‐old nymphs and adults (GLM; *df* = 1, *χ* ^2^ = 52.84, *p* < 0.001 for 9–12‐day‐old nymphs; *df* = 1, *χ* ^2^ = 13.55, *p* < 0.001 for adults; Figure 9b).

### 3.3 Insecticide resistance of *Laodelphax striatellus* with different infection states

LD_50_ values and slopes of regression lines *b* of *L. striatellus* lines with different infection states are shown in Table 1. Significant differences were not found in BPMC resistance among the four infection states. Imidacloprid resistance was significantly lower in the SW and W lines than in the S and NI_T‐7_ lines (likelihood ratio test of equality; *p* < 0.05). Etofenprox resistance was significantly lower in the SW and S lines than in the W and NI_T‐7_ lines. In the first dinotefuran test, LD_50_ values of the S and W lines were significantly higher and lower, respectively, than the value of the NI_T‐7_ line (likelihood ratio test of equality; *p* < 0.05), while the results were not reproduced in the second or third experiments (Table 1).

**Table 1:** LD_50_ values (µg/g) of *Laodelphax striatellus* lines with different infection states^a^, ^b^

	BPMC					Imidacloprid					Etofenprox				
	LD_50_ c			*b* d		LD_50_ c			*b* d		LD_50_ c			*b* d	
SW	235.4	a	(159.8–302.7)	3.5	a	0.15	a	(0.08–0.22)	1.9	ab	15.0	a	(12.0–19.3)	1.6	a
S	223.0	a	(183.4–263.5)	3.5	a	0.25	b	(0.16–0.38)	1.4	b	13.0	a	(10.1–16.3)	2.0	a
W	237.6	a	(184.7–286.4)	3.2	a	0.18	a	(0.11–0.27)	1.7	a	24.3	b	(18.7–32.4)	1.9	a
NI_T‐7_	292.0	a	(239.2–355.3)	2.7	a	0.23	b	(0.13–0.37)	1.5	b	34.2	b	(24.6–54.0)	1.3	a

	Dinotefuran 1^e^					Dinotefuran 2^e^					Dinotefuran 3^e^				
	LD_50_ c			*b* d		LD_50_ c			*b* d		LD_50_ c			*b* d	
SW	2.89	a	(2.29–3.64)	1.7	a	1.55	a	(0.61–2.50)	1.7	a	1.77	a	(1.33–2.22)	1.9	a
S	5.43	b	(2.86–17.26)	2.1	a	1.78	a	(1.36–2.21)	1.9	a	2.07	a	(1.03–3.33)	2.5	a
W	1.58	c	(1.21–1.97)	2.3	a	2.90	b	(1.94–3.92)	1.8	a	2.24	a	(1.75–2.76)	2.1	a
NI_T‐7_	2.50	a	(1.60–3.84)	2.3	a	2.87	b	(1.82–4.12)	2.4	a	2.01	a	(0.96–3.33)	1.7	a

a LD_50_ values (95% confidence intervals) in µg/g and slopes of regression lines (*b*) are shown.

b All LD_50_ values were determined 24 hr after treatment.

c The same letters indicate no significant differences between regression lines of populations by likelihood ratio tests of equality (slopes and intercepts; *p* > 0.05).

d The same letters indicate no significant differences between regression lines of populations by likelihood ratio tests of equality (slopes; *p* > 0.05).

e The resistance test of dinotefuran was performed three times.

## 4 DISCUSSION

Influences of symbionts on their hosts are categorized as either beneficial (mutualism), neutral (commensalism), or harmful (parasitism). The prosperity of insects is often facilitated by symbionts contributing to the improved fitness of their hosts (Brownlie & Johnson, 2009). The symbionts, including *Wolbachia*, that manipulate host reproduction are regarded as “selfish” genetic factors and are called “reproductive parasites” (Werren & O'Neill, 1997). In contrast, some *Wolbachia* lack these abilities and act as commensalists instead (Bordenstein & Werren, 2000; Harcombe & Hoffmann, 2004; Hoffmann, Clancy, & Duncan, 1996), in which reasons why the infected populations spread in nature are unknown, or even as mutualists (Brownlie et al., 2009; Hosokawa, Koga, Kikuchi, Meng, & Fukatsu, 2010). To confirm the fitness consequences of *Wolbachia* and *Spiroplasma* infections on *L. striatellus*, we compared the development and reproduction of *L. striatellus* with different infection states. In Group 1, the fertility of the S line was lower than that of the NI line, while significant differences between the SW and NI lines were not found. In Group 2, no significant differences were detected on their host development and reproduction. These results suggest that *Spiroplasma* infection negatively affects the reproduction of their host, but that the negative influence was relieved when *Wolbachia* coexists with *Spiroplasma*. In addition, *Wolbachia* may be only a “reproductive parasite,” which does not negatively affect the development and reproduction of its host.

We presumed that the relief of negative effect of *Spiroplasma* in coexistence with *Wolbachia* resulted from lower *Spiroplasma* density in the double‐infection state than in the single‐infection state; thus, we quantified and compared the abundance of *Spiroplasma* between the SW and S lines. Although the SW line harbored *Spiroplasma* more abundantly than the S line in early‐ and middle‐stage nymphs (3–6 and 6–9‐day‐old), the abundance of *Spiroplasma* became lower than in the S line in late‐stage nymphs (9–12‐day‐old) and adults. The lower abundance of *Spiroplasma* at late development stages supports our hypothesis that the relief of negative effect is involved in the relatively low density of *Spiroplasma* under the presence of *Wolbachia*.

The mechanism underlying the different trends in *Spiroplasma* abundance between the SW and S lines is unknown, but the lower abundance of *Spiroplasma* in the SW line in late developmental stages may be caused by the competition between the two endosymbionts. A previous study suggested that the late male‐killing of *L. striatellus* caused by *Spiroplasma* depends on the abundance of the endosymbiont because both male mortality and abundance of *Spiroplasma* became significantly higher in the later nymphal stage (Sanada‐Morimura et al., 2013). Other studies reported that bacterial densities are positively correlated with the intensity of male‐killings by endosymbionts (Anbutsu & Fukatsu, 2003; Hurst, Johnson, Schulenburg, & Fuyama, 2000). In mosquito–microsporidian relationships, a selective benefit of late male‐killing rather than early is proposed as the horizontal transmission of endosymbionts (Hurst & Majerus, 1993); however, it seems not to occur in our system. Considering the reallocation of resources, which is one of the benefits of male‐killing, male‐killing as early as possible will be desirable for *Spiroplasma*. If the male‐killing is delayed by the competition between two symbionts, the existence of *Wolbachia* may also be a disadvantage for *Spiroplasma*. This symbiotic relationship among the host and endosymbionts seems to be exclusively antagonistic.

Previous studies have reported that endosymbionts affect the insecticide susceptibility of their hosts, for example, the stinkbug *Riptortus pedestris* (Kikuchi et al., 2012), sweet potato whitefly *Bemisia tabaci* (Ghanim & Kontsedalov, 2009; Kontsedalov et al., 2008), common house mosquito *Culex pipiens* (Berticat, Rousset, Raymond, Berthomieu, & Weill, 2002), and oriental fruit fly *Bactrocera dorsalis* (Cheng et al., 2017). In this study, no positive influence of *Spiroplasma* infection was detected in the resistance to BPMC, imidacloprid, or etofenprox. Rather, *Wolbachia* and *Spiroplasma* infections negatively affected the imidacloprid and etofenprox resistances, respectively. Although the influence of *Spiroplasma* infection was positive in the first dinotefuran test, the LD_50_ value was only approximately two times higher than in the NI_T‐7_ line. Because the difference was much smaller than that of normal resistant and susceptible strains in rice planthoppers (Matsumura et al., 2014), we conducted the second and third dinotefuran tests to verify the reproducibility of this small but significant differences. As a result, these additional tests, both with rather narrow 95% confidence intervals compared to the first test, did not show a similar tendency. Field populations of *L. striatellus*, which are regarded susceptible to dinotefuran, exhibit similar LD_50_ values to the current study (1.4–4.6 µg/g; Otuka et al., 2010); thus, we concluded that *Spiroplasma* infection does not critically contribute to the development of dinotefuran resistance. However, in a previous study, *Wolbachia* infection in *L. striatellus* was suggested to be involved in the susceptibility to buprofezin, an insect growth regulator (IGR; Li, Liu, & Guo, 2018). Further studies are required to investigate the influence of *Spiroplasma* infection on susceptibility to IGRs.

No positive influences of *Spiroplasma* on the nymphal duration, adult longevity, fecundity, or insecticide resistance of *L. striatellus* were found in this study. These results suggest that *Spiroplasma* is a parasitic endosymbiont, at least in terms of host growth, development, reproduction, and focal insecticide resistance. However, positive influences of endosymbionts have been reported on other traits in aphids, for example, increasing fecundity under heat stress (Montllor, Maxmen, & Purcell, 2002) and protecting hosts from parasitic wasps (Oliver, Russell, Moran, & Hunter, 2003; Xie, Vilchez, & Mateos, 2010) or fungal pathogens (Łukasik, Asch, Guo, Ferrari, & Godfray, 2013; Scarborough, Ferrari, & Godfray, 2005). In *L. striatellus*, the growth rate of the nymphs and the number of yeast‐like symbionts were reduced at high temperatures (Noda & Saito, 1979). *Spiroplasma* may be mitigating such heat effects. Furthermore, *L. striatellus* has many natural enemies, such as the parasitoid wasp *Haplogonatopus atratus* Esaki et Hashimoto (Hymenoptera: Dryinidae; Esaki & Hashimoto, 1932,1935; Kitamura, 1987; Koyama, Takayama, Mitsuhashi, & Kishino, 1988), egg parasitoids *Anagrus* spp. (Hymenoptera: Mymaridae; Esaki & Hashimoto, 1932; Ôtake, 1967, 1968), a strepsipteran parasite *Elenchus japonicus* (Esaki et Hashimoto; Strepsiptera: Elenchidae; Esaki, 1932; Kitamura, 1987; Maeta, Machita, & Kitamura, 2007), a parasitic nematode *Agamermis unka* Kaburaki et Imamura (Mermithida: Mermithidae; Imamura, 1932; Kitamura, 1987), and pathogenic fungi *Conidiobolus* sp. (Okada, 1971) and *Metarhizium anisopliae* (Lee & Hou, 1989). In addition, *Beauveria bassiana*, known as a fungal biopesticide for many insect pests, is also considered a pathogen of *L. striatellus* because the pathogenicity for the two rice planthoppers *Nilaparvata lugens* (Stål) and *Sogatella furcifera* (Horváth) were found in a previous study (Agunda, Litsinger, & Roberts, 1984). *Spiroplasma* may be protecting the host from these parasites and pathogens.

According to Hamilton (1967), the extraordinary sex ratios caused by selfish genetic elements are exceedingly unstable and will be rapidly normalized by suppressor mechanisms. Suppression genes counteracting the male‐killing caused by endosymbionts have often spread in host populations rapidly (Charlat et al., 2007; Hayashi, Nomura, & Kageyama, 2018; Mitsuhashi, Ikeda, & Muraji, 2011). In addition, a previous study reported that another phenotype of reproductive manipulation, CI, was exhibited when the male‐killing was disabled by the suppression gene in the relationship between *Hypolimnas bolina* and *Wolbachia* (Hornett et al., 2008). Similar suppression mechanisms may be also occurring in *L. striatellus*, and then, the strategy of *Spiroplasma* may be converted to another. Furthermore, theoretical models and field investigations in previous studies indicate that multiple populations harboring different types of selfish genetic elements cannot coexist sympatrically (Charlat et al., 2006; Engelstädter, Montenegro, & Hurst, 2004; Engelstädter, Telschow, & Hammerstein, 2004). However, in the case of *L. striatellus*, the two populations showing different reproductive manipulation phenomena: the CI population infected with *Wolbachia* and the male‐killing population infected with *Wolbachia* and *Spiroplasma* were coexistent around Taiwan in 2009 and 2010 (S. Sanada‐Morimura, unpublished data). It is unknown how their symbiotic relationship will change in the future. The relationship among two symbionts differing in strategy and their host must be thoroughly investigated in further study.

## CONFLICT OF INTEREST

None declared.

## AUTHOR CONTRIBUTIONS

K.Y., S.S.‐M., and M.T. designed research. K.Y., S.S.‐M., and M.T. performed research. K.Y., S.S.‐M., S.‐H.H., and M.T. contributed to the analytical tools and analyzed data. K.Y., S.S.‐M., and M.T. wrote the paper.

## Data Availability

The DOI for our data is https://doi.org/10.5061/dryad.jh050b4.
